# Cancer Stem Cells (CSCs) in Drug Resistance and their Therapeutic Implications in Cancer Treatment

**DOI:** 10.1155/2018/5416923

**Published:** 2018-02-28

**Authors:** Lan Thi Hanh Phi, Ita Novita Sari, Ying-Gui Yang, Sang-Hyun Lee, Nayoung Jun, Kwang Seock Kim, Yun Kyung Lee, Hyog Young Kwon

**Affiliations:** Soonchunhyang Institute of Medi-bio Science (SIMS), Soonchunhyang University, Asan, Republic of Korea

## Abstract

Cancer stem cells (CSCs), also known as tumor-initiating cells (TICs), are suggested to be responsible for drug resistance and cancer relapse due in part to their ability to self-renew themselves and differentiate into heterogeneous lineages of cancer cells. Thus, it is important to understand the characteristics and mechanisms by which CSCs display resistance to therapeutic agents. In this review, we highlight the key features and mechanisms that regulate CSC function in drug resistance as well as recent breakthroughs of therapeutic approaches for targeting CSCs. This promises new insights of CSCs in drug resistance and provides better therapeutic rationales to accompany novel anticancer therapeutics.

## 1. Introduction

Cancer is one of the leading causes of morbidity and mortality worldwide with about 20% of all deaths in developed countries [1]. From preclinical and clinical cancer studies, various new diagnostic and treatment options for cancer patients provide notable progresses in cancer treatment and prevention [2]. Cancer heterogeneity is one of the reasons contributing to the treatment failure and disease progression. Among several cancer treatments, the main treatments that are commonly used to treat patients are surgery, radiotherapy, and chemotherapy. Surgery can successfully remove cancer from the body, while combining radiotherapy with chemotherapy can effectively give better results for treating many types of cancer [3]. Recent chemotherapeutic agents are successful against primary tumor lesions and its residue after surgery or radiotherapy [4]. However, chemotherapy induces tumor heterogeneity derived from both normal and cancer cells and the heterogeneity within tumors, in turn, results in reducing effects of chemotherapy; contributing to the treatment failure and disease progression [5, 6]. Chemoresistance is a major problem in the treatment of cancer patients, as cancer cells become resistant to chemical substances used in treatment, which consequently limits the efficiency of chemo agents [7]. It is also often associated with tumors turning into more aggressive form and/or metastatic type [8–11].

Accumulating evidences suggest that cancer stem cell (CSC) population, a subgroup of cancer cells, is responsible for the chemoresistance and cancer relapse, as it has ability to self-renew and to differentiate into the heterogeneous lineages of cancer cells in response to chemotherapeutic agents [12–14]. CSCs are also able to induce cell cycle arrest (quiescent state) that support their ability to become resistant to chemo- and radiotherapy [15–20]. Common chemotherapeutic agents target the proliferating cells to lead their apoptosis, as mentioned previously. Although successful cancer therapy abolishes the bulk of proliferating tumor cells, a subset of remaining CSCs can survive and promote cancer relapse due to their ability to establish higher invasiveness and chemoresistance [21, 22]. Understanding the features of CSCs is important to establish the foundation for new era in treatment of cancer. In this review, we address the detailed mechanisms by which CSCs display the resistance to chemo- and radiotherapy and their implication for clinical trials.

## 2. The Origin and Surface Markers of Cancer Stem Cells (CSCs)

Cancer stem cells (CSCs), also known as tumor-initiating cells (TICs), have been intensively studied in the past decade, focusing on the possible source, origin, cellular markers, mechanism study, and development of therapeutic strategy targeting their pathway [23, 24]. The first convincing evidence of CSCs was reported by Bonnet and Dick in 1997 by the identification of a subpopulation of leukemia cells expressing surface marker CD34, but not CD38. CD34^+^/CD38^−^ subpopulation was capable of initiating tumor growth in the NOD/SCID recipient mice after transplantation [25]. In addition to blood cancer, CSCs have been identified in several kinds of solid tumor [21, 26]. The first evidence of the presence of CSCs in solid cancer in vivo was found and identified as CD44^+^CD24^-/low^Lineage^−^ cells in immunocompromised mice after transplanting human breast cancer cells in 2003 [27] even though it has been indicated in vitro in 2002 by the discovery of clonogenic (sphere-forming) cells isolated from human brain gliomas [28]. Over time, CSC population was also identified from several other solid cancers including melanoma, brain, lung, liver, pancreas, colon, breast cancer, as well as ovarian cancer [27, 29–35].

**Figure 1:**
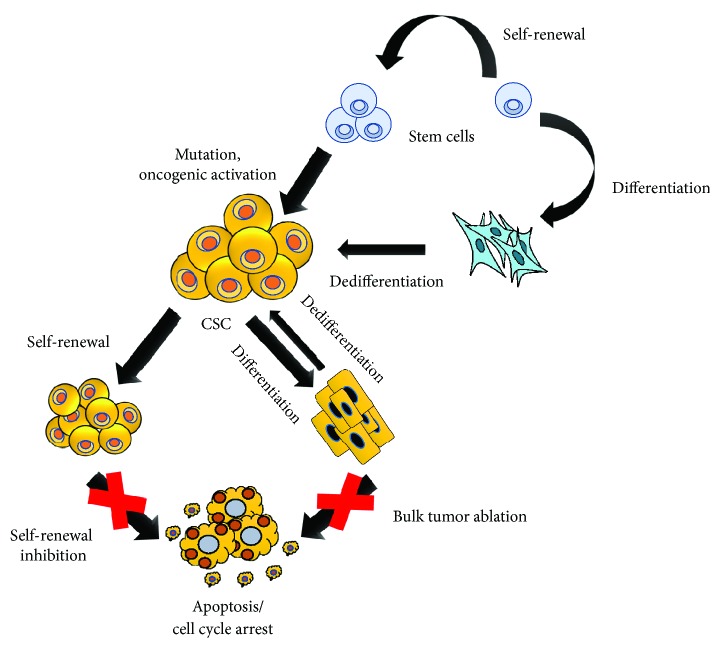
The origin of CSCs and combinational therapy of CSC targeting and bulk tumor ablation. CSCs could possibly have originated from either stem cells with mutation/oncogenic transformation, progenitors that have undergone mutation, or from differentiated cells or cancer cells that obtained stem-like properties by dedifferentiation. Thus, because of the plasticity of CSCs, it is suggested that combinational therapy of CSC targeting and bulk tumor ablation may have better therapeutic effects to improve the clinical outcomes of cancer patients.

Although CSC model explains the heterogeneity of cancers in terms of hierarchical structure and progression mode, the origins of CSCs are currently unclear and controversial [36, 37]. Accumulating hypotheses suggest that depending on the tumor type, CSCs might be derived from either adult stem cells, adult progenitor cells that have undergone mutation, or from differentiated cells/cancer cells that obtained stem-like properties through dedifferentiation [25, 38–50]. Because of the plasticity of CSCs, it has been suggested that the combinational therapy of targeting CSC pathways and conventional chemotherapeutics might have better therapeutic effect, which will be explained later in detail (Figure 1). Early studies in AML demonstrated that normal primitive cells, but not committed progenitor cells, are targets for leukemic transformation [25]. Similarly, it has been indicated that deletion of Apc in Lgr5^+^ (leucine-rich-repeat containing G-protein coupled receptor 5) long-lived intestinal stem cells, rather than short-lived transit-amplifying cells, could lead to their transformation, showing that stem cells are the cells-of-origin in intestinal cancer [42]. Moreover, long-term culture can also induce telomerase-transduced adult human mesenchymal stem cells (hMSCs) to undergo spontaneous transformation, showing that these cells are also the origin of CSCs [43, 44]. Interestingly, CSCs originate from the transformation of not only their tissue-specific stem cells but also other tissue stem cells. For instance, bone marrow-derived cells (BMDCs) may be an essential source of many tumor types, such as gastric cancer, neural tumors, and even teratoma [45].

CSCs also have been demonstrated to be generated by dedifferentiation from progenitor cells or differentiated cells which have acquired “stemness” properties as a result of the accumulation of extra genetic or epigenetic abnormalities [46]. For example, BCR-ABL fusion protein is present in hematopoietic stem cell- (HSC-) like CML cells but granulocyte-macrophage progenitors are found to be a candidate of the advanced-stage LSCs during blast crisis in blast-crisis CML by activating the self-renewal process via *β*-catenin pathway [47]. In addition, it has been shown that oncogenic Hh signaling can promote medulloblastoma from either lineage-restricted granule cell progenitors or stem cells [48, 49]. Besides, most differentiated cells in the CNS, including terminally differentiated neurons and astrocytes, can acquire defined genetic alterations to dedifferentiate into NSC or progenitor state and consequently induce and maintain malignant gliomas [50].

Of note, CSCs can be identified by specific markers of normal stem cells which are commonly used for isolating CSCs from solid and hematological tumors [51]. Several cell surface markers have been verified to identify CSC-enriched populations, such as CD133, CD24, CD44, EpCAM (epithelial cell adhesion molecule), THY1, ABCB5 (ATP-binding cassette B5), and CD200 [27, 32, 34, 52]. Additionally, certain intracellular proteins also have been used as CSCs markers, such as aldehyde dehydrogenase 1 (ALDH1) which is used to characterize CSCs in many types of cancer such as leukemia, breast, colon, liver, lung, pancreas, and so forth [12, 53]. The usage of cell surface markers as CSC markers might differ from each cancer types depending on their characteristics and phenotypes. The surface markers that are frequently used to isolate CSCs from each cancer types are listed in Table 1.

## 3. The Mechanisms by Which CSCs Contribute to the Resistance against Chemotherapy and Cancer Relapse

**Figure 2:**
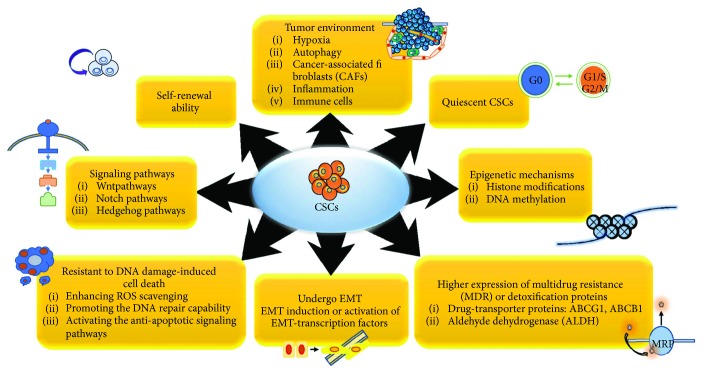
Key signaling pathways and modifications of CSCs contributing to the resistance against chemotherapeutics. In order to survive during and after therapy, CSCs display many responses including EMT, self-renewal, tumor environment, quiescence, epigenetic modification, MDR, and so forth. The mechanisms by which CSCs contribute to resistance against therapeutics are summarized.

Recent studies suggest that CSCs are enriched after chemotherapy because a small subpopulation of cells remaining in tumor tissue, so-called CSCs, can survive and expand though most chemotherapeutic agents kill bulk of tumors [12–14]. For instance, preleukemic DNMT3Amut HSCs which can initiate clonal expansion as the first step in leukemogenesis and regenerate the entire hematopoietic hierarchy were found to survive and expand in the bone marrow remission after chemotherapy [54]. Similarly, exposure to therapeutic doses of temozolomide (TMZ), the most commonly used antiglioma chemotherapy, consistently expands the glioma stem cell (GSC) pool over time in both patient-derived and established glioma cell lines, which has been shown to be a result of phenotypic and functional interconversion between differentiated tumor cells and GSCs [55]. Moreover, the humanized VEGF antibody bevacizumab reduces glioblastoma multiforme (GBM) tumor growth but followed by tumor recurrence, possibly due to the ongoing autocrine signaling through the VEGF-VEGFR2–Neuropilin-1 (NRP1) axis, which is associated with the enrichment of active VEGFR2 GSC subset in human GBM cells [56]. The gefitinib-resistant subline HCC827-GR-highs established by high-concentration method also acquire both the EMT and stem cell-like features but do not show any EGFR-mutant–specific protein production or further increase in the number of either mutant allele or EGFR copy [57]. Therefore, by understanding the mechanisms and oncogenic drivers by which the CSCs escape the radio- and chemotherapy, we can develop more effective treatments that could improve the clinical outcomes of cancer patients. The mechanisms by which CSCs contribute to the chemoresistance including EMT, MDR, dormancy, tumor environment, and so forth are mentioned below in detail and summarized in Figure 2.

### 3.1. Epithelial Mesenchymal Transition (EMT)

It has been indicated that epithelial mesenchymal transition (EMT) markers and stem cell markers are coexpressed in circulating tumor cells from patients with metastasis [58] and EMT induction or activation of EMT transcription factors (TFs) confers stem-like features in cancer cells [59]. In particular, normal and neoplastic human breast stem-like cells express similar markers with cells that have undergone EMT, and EMT induces the generation of relatively unlimited numbers of cancer stem cells from more differentiated neoplastic cells [60]. Meanwhile, there is an association between EMT activation and drug resistance [61]. For instance, gefitinib-induced resistant lung cancer cells acquire EMT phenotype [62] through activation of Notch-1 signaling [63]. Moreover, enhanced invasive potential, tumorigenicity, and expression of EMT markers could be used to predict the resistance of anti-EGFR antibody cetuximab in the cells [64]. In parallel, compared with epithelial cell lines, the mesenchymal cells have increased expression of genes involved in metastasis and invasion and are significantly less susceptible to EGFR inhibition, including erlotinib, gefitinib, and cetuximab; at least partly via integrin-linked kinase (ILK) overexpression in mesenchymal cells [65]. Besides, EMT mediator S100A4 has been shown to involve in maintaining the stemness properties and tumorigenicity of CSCs in head and neck cancer [66]. Therefore, EMT induces the cancer cells to exhibit stem cell-like characteristics which promote cells to invade surrounding tissues and display therapeutic resistance [67]. Interestingly, ZEB1, a regulator of EMT, plays an important role in key features of cancer stem cells including the regulation of stemness and chemoresistance induction through transcriptional regulation of O-6-Methylguanine DNA Methyltransferase (MGMT) via miR-200c and c-MYB in malignant glioma [68]. Apart from EMT, the high expression of stem cell markers such as Oct4, Nanog, Sox2, Musashi, and Lgr5 has been considered to confer chemoresistance as well [69–73].

### 3.2. High Levels of Multidrug Resistance (MDR) or Detoxification Proteins

Side population (SP) cells, which exhibit a cancer stem cell-like phenotype, are detected in a variety of different solid tumors such as retinoblastoma, neuroblastoma, gastrointestinal cancer, breast cancer, lung cancer, and glioblastoma; their high expression of drug-transporter proteins (including MDR1, ABCG2, and ABCB1) not only acts to exclude Hoechst dye but also expels cytotoxic drugs, leading to high resistance to chemotherapeutic agents with better cell survival and disease relapse [74–76]. Alisi et al. suggest that the overexpression of ABC protein is probably the most important protective mechanism for CSCs in response to chemotherapeutic agents [77]. Interestingly, it has been demonstrated that the PI3K/Akt pathway specifically regulates ABCG2 activity via its localization to the plasma membrane, and loss of PTEN promotes the SP phenotype of human glioma cancer stem-like cells [78]. Moreover, the activity of aldehyde dehydrogenase (ALDH), a cytosolic enzyme that is responsible for the oxidation of intracellular aldehydes to protect cells from the potentially toxic effects of elevated levels of reactive oxygen species (ROS) [79], is high in both normal and patients' CD34^+^/CD38^−^ leukemic stem cells, and thus plays an important role in resistance to chemotherapy [80]. ALDH activity is a potential selective marker for cancer stem cells in many different types of cancer, such as breast cancer [53], bladder cancer [81], head and neck squamous cell carcinoma [82], lung cancer [83], and embryonal rhabdomyosarcoma [84]. Interestingly, cell culture model systems and clinical sample studies show that ALDH1A1-positive cancer stem cells promote significant resistance to both EGFR-TKI (gefitinib) and other anticancer chemotherapy drugs (cisplatin, etoposide, and fluorouracil) than ALDH1A1-negative cells in lung cancer [85]. In addition, high levels of ALDH1 expression predict a poor response or resistance to preoperative chemoradiotherapy in resectable esophageal cancer patients [86].

### 3.3. Dormancy of CSCs

It has been demonstrated that besides the intratumoral heterogeneity initiated by the evolution of genetically diverse subclones, there are also functionally distinct clones, which were found by tracking the progeny of single cells using lentivirus, within a genetic lineage in colorectal cancers [87]; accordingly, these diversity-generating processes within a genetic clone promote cells for higher survival potential, especially during stress such as chemotherapy. For example, chemotherapy can induce the tumor growth of previously relatively dormant or slowly proliferating lineages that still retain potent tumor propagation potential, leading to both cancer growth and drug resistance [87]. Similarly, in glioblastoma multiforme, there is also the existence of a relatively quiescent subset of endogenous tumor cells with characteristics similar to cancer stem cells responsible for maintaining the long-term tumor growth and therefore leading to recurrence via the production of transient populations of highly proliferative cells [17]. Concomitantly, chemotherapy-induced damages recruit the quiescent label-retaining pool of bladder CSCs during the gap periods between chemotherapy cycles into an unexpected cell division response to repopulate residual tumors, similar to wound repair mobilization of tissue resident stem cells [88].

### 3.4. Resistance to DNA Damage-Induced Cell Death

CSCs can be resistant to DNA damage-induced cell death through several ways. These include protection against oxidative DNA damage by enhanced ROS scavenging, promotion of the DNA repair capability through ATM and CHK1/CHK2 phosphorylation, or activation of the anti-apoptotic signaling pathways, such as PI3K/Akt, WNT/b-catenin, and Notch signaling pathways [24]. For instance, CD44, an adhesion molecule expressed in CSC, interacts with a glutamate-cystine transporter and controls the intracellular level of reduced glutathione (GSH); hence, the CSCs expressing a high level of CD44 showed an enhanced capacity for GSH synthesis, resulting in stronger defense against ROS [89]. Interestingly, similar to normal tissue stem cells, CSCs have lower ROS levels, which is associated with increased expression of free radical scavenging systems, leading to higher ROS defenses and radiotherapy resistance as well [90]. In addition, CD44^+^/CD24^−/low^ CSC subset in breast cancer is resistant to radiation via activation of ATM signaling but does not depend on alteration of nonhomologous end joining (NHEJ) DNA repair activity [91]. Similarly, CD133-expressing tumor cells isolated from both human glioma xenografts and primary patient glioblastoma specimens preferentially activate the DNA damage checkpoint in response to radiation and repair radiation-induced DNA damage more effectively than CD133-negative tumor cells [92]. Notch pathway also promotes the radioresistance of glioma stem cells as the Notch inhibition with gamma-secretase inhibitors (GSIs) induces the glioma stem cells to be more sensitive to radiation at clinically relevant doses due to the reduction of radiation-induced PI3K/Akt activation and upregulated levels of truncated apoptotic isoform of Mcl-1 (Mcl-1s) while not altering DNA damage response [93].

### 3.5. Tumor Environment

It has been shown that a distinct microenvironment of various cellular composition is important to protect and regulate normal stem cells. An equivalent microenvironment was also found in the CSCs in which CSCs was favorably supported within a histologic niche, so-called CSC microenvironment [94–96], containing connective stromal [97–101] and vascular tissues [102–106]. This environment expedites CSC divisional dynamics, allowing them to differentiate progenitor daughter cells as well as self-renew and maintain CSCs in the primitive developmental state. The cells within CSC microenvironment are capable of stimulating signaling pathways [58], such as Notch [102, 107, 108] and Wnt [109–111] which may facilitate CSCs to metastasize, evade anoikis, and alter divisional dynamics, achieving repopulation by symmetric division [109, 112–114].

#### 3.5.1. Hypoxia

Hypoxia and HIF signaling pathway have been shown to contribute to the regulation and sustenance of CSCs and EMT phenotype such as cell migration, invasion, and angiogenesis [115], via the increased expression of VEGF, IL-6, and CSC signature genes such as Nanog, Oct4, and EZH2, in pancreatic cancer for example [116]. Therefore, hypoxia and HIF signaling pathway may also play a role in CSC resistance to therapy. In the hypoxic microenvironment, hypoxia and hypoxia-inducible factor HIF1-*α* signaling promote CML cell persistence mainly through the upregulation of hypoxia-inducible factor 1*α* (HIF1-*α*), independent of BCR-ABL1 kinase activity [117]. Similarly, hypoxia increases gefitinib-resistant lung CSCs in EGFR mutation-positive NSCLC by upregulating expression of insulin-like growth factor 1 (IGF1) through HIF1*α* and activating IGF1 receptor (IGF1R) [118]. Interestingly, autophagy is upregulated in the pancreatic cancer in the microenvironmental condition of low oxygen and lack of nutrition, similar with the hypoxic tumor, and then promotes the clonogenic survival and migration of pancreatic CSC^high^ cells [119].

#### 3.5.2. Cancer-Associated Fibroblasts (CAFs)

It has been indicated that besides cell autonomous resistance of CSCs, chemotherapy preferentially targets non-CSCs by the stimulation of cancer-associated fibroblasts (CAFs) which creates a chemoresistant niche by increased secretion of specific cytokines and chemokines, including interleukin-17A (IL-17A), a CSC maintenance factor by promoting self-renewal and invasion [120]. It has been shown previously that CSCs can differentiate into CAF-like cells (CAFLCs) and hence they are one of the key sources of CAFs which support the tumor maintenance and survival in the cancer niche [121]. CAFs are known to secrete many different growth factors, cytokines, and chemokines, including hepatocyte growth factor (HGF), which activates the MET receptors to protect the CSCs from apoptosis in response to the cetuximab monotherapy targeting the EGFR in metastatic colorectal cancer [122].

#### 3.5.3. Inflammation

In addition, long-term treatment of breast cancer cells with trastuzumab specifically enriched CSCs which exhibit EMT phenotypes with higher levels of secreted cytokines IL-6 compared with parental cells; as a consequence, these cells develop trastuzumab resistance mediated by activation of an IL-6-mediated inflammatory feedback loop to expand the CSC population [123]. Similarly, autocrine TGF-*β* signaling and IL-8 expression are also enhanced after chemotherapeutic drug paclitaxel treatment in triple-negative breast cancer, leading to CSC population enrichment and tumor recurrence [124]. Furthermore, stroma-secreted chemokine stroma-derived factor 1a (SDF-1a) and its cognate receptor CXCR4 play an important role in the migration of hematopoietic cells to the bone marrow microenvironment [125, 126], so SDF-1A/CXCR4 interaction mediates the resistance of leukemia cells to chemotherapy-induced apoptosis [127], and thus CXCR4 inhibition with inhibitors such as AMD3100 can enhance the sensitivity of leukemic cells to chemotherapy by disrupting stromal/leukemia cell interactions within the bone marrow microenvironment by Akt phosphorylation inhibition and PARP cleavage induction due to bortezomib in the presence of bone marrow stromal cells (BMSCs) in coculture [128]. Moreover, the CSCs from the chemoresistant tumors have the unique ability to produce a variety of proinflammatory signals, such as IFN regulatory factor-5 (IRF5), which acts as a transcription factor specific for chemoresistant tumors to induce the M-CSF production, to consequently produce the M2-like immunoregulatory myeloid cells from CD14^+^ monocytes, and to promote the myeloid cell-mediated tumorigenic and stem cell activities of bulk tumors [129].

#### 3.5.4. Immune Cells

It has been indicated previously that tumor-associated macrophages (TAMs) can promote chemoresistance in both myeloma cell lines and primary myeloma cells from spontaneous or chemotherapeutic drug-induced apoptosis by directly interacting with malignant cells within the tumor microenvironment and attenuating the activation and cleavage of caspase-dependent apoptotic signaling [130]. Moreover, TAM also directly induces CSC properties of pancreatic tumor cells by activating signal transducer and activator of transcription 3 (STAT3) and thus inhibits the antitumor CD8^+^ T lymphocyte responses in the chemotherapeutic response [131]. Besides, in pancreatic ductal adenocarcinoma, cancer cells secrete colony-stimulating factor 1 (CSF1) to attract and stimulate CSF1 receptor- (CSF1R-) expressing TAM to express high levels of cytidine deaminase (CDA), an intracellular enzyme which catabolizes the bioactive form of gemcitabine and therefore protects the cancer cells from the chemotherapy [132].

### 3.6. Epigenetics

Besides, CSC-mediated drug resistance is regulated by epigenetic mechanisms as well, including histone modifications and DNA methylation. First, DNA methylation was unchanged during TGF-*β*-mediated EMT but other epigenetic changes such as a lysine-specific deaminase-1- (Lsd1-) dependent global reduction of the heterochromatin mark H3-lys9 dimethylation (H3K9Me2), an increase of the euchromatin mark H3-lys4 trimethylation (H3K4Me3) and the transcriptional mark H3-lys36 trimethylation (H3K36Me3) are found; especially, H3K4Me3 might contribute to Lsd1-regulated chemoresistance [133]. In addition, KDM1A, a flavin adenine dinucleotide- (FAD-) dependent lysine-specific demethylase specifically with monomethyl- and dimethyl-histone H3 lysine-4 (H3K4) and lysine-9 (H3K9) substrate, is an important regulator of MLL-AF9 leukemia stem cell (LSC) oncogenic potential by blocking differentiation [134]. Besides, B-cell-specific Moloney murine leukemia virus integration site 1 (BMI1), one of several epigenetic silencer proteins belonging to Polycomb group (PcG), is required for self-renewal of both adult stem cells and many CSCs via various key pathways, such as anchorage-independent growth, Wnt and Notch pathway [135]. BMI-1 has been indicated to be involved in the protection of cancer cells from apoptosis or drug resistance in various types of cancer, including nasopharyngeal carcinoma [136], melanoma [137], pancreatic adenocarcinoma [138], ovarian cancer [139], and hepatocellular carcinoma [140]. Furthermore, another PcG member EZH2, a catalytic subunit of polycomb repressor complex 2 (PRC2) which trimethylates histone H3 at lysine 27 (H3K27me) and elicits gene silencing, also participates in pancreatic cancer chemoresistance by silencing p27 tumor suppressor gene via methylation of histone H3-lysine 27 (H3K27) [141]. Moreover, EZH2 protects GSCs from radiation-induced cell death and consequently promotes GSC survival and radioresistance via upstream regulator mitotic kinase maternal embryonic leucine-zipper kinase (MELK) [142]. In addition, EZH2 inhibition sensitizes BRG1 and EGFR loss-of-function mutant lung tumors to topoisomerase II (TopoII) inhibitor etoposide with increased S phase, anaphase bridging, and apoptosis [143]. Interestingly, EZH2 and BMI1 are indicated to inversely correlate with prognosis signature and TP53 mutation in breast cancer [144].

Second, histone acetylation is involved in the regulation of transcriptional activation and chemoresistance of CSCs too. Treatment with HDAC inhibitors (HDACi) effectively targets the quiescent chronic myelogenous leukemia (CML) stem cells which are resistant to tyrosine kinase inhibitor imatinib mesylate (IM) [145]. Similarly, pretreatment with HDAC inhibitors may sensitize the prostate stem-like cells to radiation treatment through increased DNA damage and reduced clonogenic survival [146]. Vorinostat, a HDAC inhibitor via inducing ubiquitination and lysosome degradation, downregulates the expression and signaling of all three receptors EGFR, ErbB2, and ErbB3 together with reversion of EMT in EGFR TKI gefitinib-resistant cells and therefore enhances the antitumor effect of gefitinib in squamous cell carcinoma of head and neck [147]. Interestingly, NANOG upregulates histone deacetylases 1 (HDAC1) via binding to the promoter region and decreasing K14 and K27 histone H3 acetylation; as a result, it induces not only the stem-like features through epigenetic repression of cell cycle inhibitor CDKN2D and CDKN1B but also the immune resistance and chemoresistance through MCL-1 upregulation by epigenetic silencing of E3 ubiquitin-ligase TRIM17 and NOXA [148].

Third, many tumor suppressor genes have been shown to be epigenetically silenced in chemoresistant cancers by DNA methylation on CpG promoter regions. For instance, tumor suppressor insulin-like growth factor binding protein-3 (IGFBP-3), which is involved in controlling cell growth, transformation, and survival, is specifically downregulated through promoter-hypermethylation and results in acquired resistance to chemotherapy in many different types of cancer [149]. In addition, loss of DNA mismatch repair (MMR) gene hMLH1 via full hypermethylation of the hMLH1 promoter [150] is highly correlated with the ability of arresting cell death and cell cycle after DNA damage induced by chemotherapy and poor survival prediction for cancer patients [151], hence plays a role in drug resistance in ovarian [152] and breast cancers [153].

### 3.7. Signaling Pathways of CSC-Driven Chemoresistance

As mentioned, normal stem cells and CSCs have similar characteristics such as self-renewal and differentiation. They also share numbers of key signaling pathway to maintain its existence. For example, Notch signaling was highly expressed in the hematopoietic tumors such as T-ALL and solid tumors such as non-small-cell lung carcinoma (NSCLC), breast cancer, and glioblastoma [154–156]. Activation of Hedgehog signaling which in normal condition plays important roles in embryonic development and tissue regeneration also has been found to be involved in the regulation of various cancer stem cells, such as pancreatic cancer, leukemias, and basal cell carcinoma (BCC) [157]. Another signaling pathway such as WNT, TGF*β*, PI3K/Akt, EGFR, and JAK/STAT, as well as transcriptional regulators including OCT4, Nanog, YAP/TAZ, and Myc are also commonly activated in various cancer stem cells to regulate their self-renewal and differentiation state [21, 158]. CSCs have been indicated to display many characteristics of embryonic or tissue stem cells and developmental signaling pathways such as Wnt, HH, and Notch that are highly conserved embryonically and control self-renewal of stem cells [159]. Therefore, activation of these pathways may play an important role in the expansion of CSCs and hence the resistance to therapy [160]. Here, several representatives are explained.

First, it has been indicated that activation of Wnt/*β*-catenin signaling enhances the chemoresistance to IFN-*α*/5-FU combination therapy [161]. OV6^+^ HCC cells, a subpopulation of less differentiated progenitor-like cells in HCC cell lines and primary HCC tissues, have been shown to be endogenously active Wnt/*β*-catenin signaling and resistant to standard chemotherapy [162]. In addition, in neuroblastoma, amplification and upregulation of frizzled-1 Wnt receptor (FZD1) activate the Wnt/*β*-catenin pathway in chemoresistant cancer cells by nuclear *β*-catenin translocation and transactivation of Wnt target genes such as multidrug resistance gene (MDR1), which is known to mediate the resistance to chemotherapy [163]. Furthermore, c-Kit, a stem cell factor (SCF) receptor, mediates chemoresistance through activation of Wnt/*β*-catenin and ATP-binding cassette G2 (ABCG2) pathway in ovarian cancer [164].

Secondly, Hh pathway could regulate autophagy in CML cells and then inhibition of the Hh pathway and autophagy simultaneously could sharply reduce cell viability and significantly induce apoptosis of imatinib-sensitive or -resistant BCR-ABL^+^ cells via downregulating the kinase activity of the BCR-ABL oncoprotein [165]. Concomitantly, the expression of sonic hedgehog (SHH) and glioma-associated oncogene homolog 1 (GLI1), the well-known signaling pathway molecules involved in the drug resistance, is higher in enriched CD44^+^/Musashi-1^+^ gastric cancer stem cells and consequently enhances the drug resistance via high drug efflux pump activity [166]. In glioma, CD133^+^ CSC population, which contributes to the chemoresistance of therapy such as temozolomide (TMZ) treatment, overexpresses genes involved in Notch and SHH pathways and activates these pathways [167].

Last but not least, chemotherapy such as oxaliplatin induces Notch-1 receptor and its downstream target Hes-1 activity by increasing gamma-secretase activity in colon cancer cells; hence, inhibition of Notch-1 signaling by gamma-secretase inhibitors (GSIs) sensitizes colon cancer cells to chemotherapy [168]. Moreover, Notch signaling pathway and Notch3 in particular play an essential role in the regulation of CSC maintenance and chemoresistance to platinum in ovarian cancer therapy [169]. Similarly, the enrichment of CD133^+^ cells in lung adenocarcinoma after cisplatin induction leads to multidrug resistance through activation of Notch signaling as higher levels of cleaved Notch1 (NICD1) are detected [170]. Furthermore, it has been shown that gefitinib-acquired resistant lung adenocarcinoma cells undergo EMT by activation of Notch-1 signaling via Notch-1 receptor intracellular domain (N1IC), the activated form of the Notch-1 receptor [63].

Besides, there are also some molecules which act as the integration of various pathways involved in the control of stem cell fate across tissues; for example, CYP26, a primary retinoid-inactivating enzyme through retinoid and Hedgehog pathways, limits the retinoic acid concentration, therefore leading to drug resistance in the stem cell niche [171].

## 4. CSC-Based Therapy

Owing to the ability of CSCs to develop chemo- and radioresistance which play key roles in the malignant progression, metastasis, and cancer recurrence, it is suggested that targeting cancer stem cells offers an ultimate goal to overcome a poor prognosis, leading to a better patient survival [15, 22]. Selective targeting of CSC signaling networks that are essential for self-renewal, proliferation, and differentiation to maintain their stem cell properties provides a new challenge in the development of cancer treatments [19, 172]. Over the last decades, it was suggested that the combination of conventional therapy and targeted therapy against CSC-specific pathways gives rise a better consequence compared to monotherapy in removal of both bulk tumor and CSC population (Figure 1) [19]. Thus, targeting essential pathways in the CSCs such as Notch, Wnt, and Hedgehog (HH) is being developed to block the self-renewal of CSCs [21]. Lately, some classes of Notch pathway inhibitors have been reported to enter a clinical trial, accompanied by a substantial variety of targets, mechanism of action, and drug classes [19, 21]. The major class of Notch inhibitor is the *γ*-secretase inhibitors (GSIs). GSI works by inhibiting the final proteolytic cleavage of Notch receptors, which results in the release of the active intracellular fragment. It was the first class of Notch pathway inhibitor that enters a clinical trial in the cancer field [159, 173, 174].

HH pathway is shown to be involved in several essential developmental pathways such as tissue patterning during embryonic development and the repair of normal tissues and epithelial-to-mesenchymal transition [175]. Vismodegib, a drug targeting HH pathway, was approved by the European Medicines Agency (EMA) in 2013 and the US FDA in 2012 for the therapy of metastatic BCC patients or locally advanced BCC patients that are not candidates for surgery or radiotherapy [176, 177].

Targeting Wnt signaling has also shown promising results related to carcinogenesis, tumor invasiveness, and metastasis [159]. Wnt3A-neutralizing mAb was shown to have antiproliferation and proapoptotic effects in prostate cancer mouse model [178]. And anti-Fz10 radio-labeled mAb is being evaluated in a phase I trial for the synovial sarcoma therapy. Vantictumab (OMP-18R5, a mAb that blocks five Fz receptors such as Fz1, Fz2, Fz5, Fz7, and Fz8) [179–181] and OMP-54F28 [181] (a mAb that blocks fusion protein decoy receptor such as truncated Fz8) are under investigation in phase I studies in advanced-stage solid tumors [182].

Targeting CSCs through the EMT pathways also provides a new challenge in the cancer therapy study. This therapy is developed in order to prevent cancer aggressiveness and acquired drug resistance of cancer stem cells [183, 184]. Lately, the finding of therapeutic agents to EMT-based CSC therapy indicated three general target groups [184, 185]. These include a group involved in the regulation of EMT extracellular inducer such as TGF-*β*, EGF, Axl-Gas6 pathways, hypoxia, and extracellular matrix components. Another group is the transcription factors (TFs) that promote EMT transcriptome including Twist1, Snail1, Zeb1/2, T-box TF Brachyury as well as its downstream effectors of EMT, such as E-Cadherin, N-Cadherin, vimentin, and HoxA9. The last one is targeting regulators of EMT-TFs and epigenetic regulator using microRNA [184–190].

Accumulating evidence suggests that miRNA and other groups of long noncoding RNA (lncRNA) play important roles in the regulation of CSCs properties such as self-renewal, asymmetric cell division, tumor initiation, drug resistance, and disease recurrence [186, 187, 189, 191–193].The usage of miRNA as CSC-based therapeutic agents is reported; for example, mir-22 that targets TET2 in leukemia (AML and MDS) and breast cancer [194], Let-7 to target RAS and HMGA2 in breast cancer [195], mir-128 to target BMI-1 in brain cancer [191], mir-200 to target ZEB1/ZEB2, BMI-1, and SUZ12 in breast cancer [189, 196, 197], and some other miRNA in the colon cancer and prostate cancer have been reported to reduce cancer malignancy [198–202].

Finally, cancer immunotherapy may be a breakthrough for targeting specifically CSCs in cancer patients. For cancer immunotherapy, several effectors, including natural killer (NK) cells and *γδ*T cells in innate immunity, antibodies in acquired humoral immunity, CSC-based dendritic cells, and CSC-primed cytotoxic T lymphocytes (CTLs) in acquired cellular immunity, which are able to recognize and kill CSCs may be suitable candidates to improve the efficacy of cancer treatment. A variety of immunotherapeutic strategies that specifically target CSCs using these effector cells have been reported. In addition, identification of specific antigens or genetic alterations in CSCs plays an important role in finding targets for immunotherapy. These include CSC markers (ALDH [203], CD44 [204, 205], CD133 [206], EpCAM [207], and HER2 [208]), CSC niche interaction (TAM [209]), tumor microenvironment (immune cells/myeloid-derived suppressor cells), cytokines (IL1 [210], IL6 [211], and IL8 [212]), and immune checkpoint (CTLA-4 [213] or PD1/PDL1 [214]).

## 5. Conclusion

CSCs possess stem cell-like features found in cancer and have important implications for the chemoresistance and cancer relapse, a notion that remains somewhat controversial. With a small subpopulation in the malignant cell pool, the contribution of CSCs is remarkable in cancer therapy, as shown by intensive studies in recent decades. These cells can be identified based on the presence of surface biomarkers, enhanced spheroid or colony formation in vitro and augmented tumor-initiating potential as well as tumorigenic ability in vivo. They are resistance to chemotherapy and radiation therapy compared to bulk tumor cells and hence play a crucial role in tumor recurrence after anticancer therapy. To survive following cancer treatment, CSCs seem to be able to manifest several responses such as EMT, induction of signaling pathways that regulate self-renewal or influence tumor environments, expression of drug transporters or detoxification proteins, and so forth to protect them from devastating effects caused by therapeutic agents. Thus, the development of anticancer therapeutics that target CSCs is not only limited to the finding of inhibitor of CSC pathways and cell surface markers but also to the development of EMT and CSCs microenvironment-related inhibitors. Though the molecular mechanisms underlying the resistance of CSCs to chemotherapy and radiation still require further studies in order to develop promising strategies for suppressing tumor relapse and metastasis, recent technological advances made it easier than before to find mechanisms contributing to drug resistance. Also, the recent therapeutic strategy of combining molecules specifically targeting CSCs with conventional chemotherapeutic drugs could possibly be a better direction for anticancer therapy and may therefore achieve better survival rates of cancer patients (Figure 1) [19]. Besides, as some cell surface biomarkers and signaling pathways are similar between CSCs and normal stem cells, it is also essentially required to develop novel therapeutic agents targeting only CSCs to avoid off-target effects on noncancerous cells or normal stem cells.

## Figures and Tables

**Table 1:** Cancer stem cell markers in human.

Tumor type	Cancer stem cell markers	Reference
Lung cancer	CD133^+^, CD44^+^, ABCG2, ALDH, CD87^+^, SP, CD90^+^	[215–217]
Colon cancer	CD133^+^, CD44^+^, CD24^+^, CD166^+^, EpCAM^+^, ALDH, ESA	[218, 219]
Liver cancer	CD133^+^, CD44^+^, CD49f^+^, CD90^+^, ALDH, ABCG2, CD24^+^, ESA	[51, 219]
Breast cancer	CD133^+^, CD44^+^, CD24^−^, EpCAM^+^, ALDH-1	[51, 218]
Gastric cancer	CD133^+^, CD44^+^, CD24^+^	[215, 220–222]
Leukemia (AML)	CD34^+^, CD38^−^, CD123^+^	[216, 218, 223]
Prostate cancer	CD133^+^, CD44^+^, *α*2*β*1, ABCG2, ALDH	[51, 215, 223]
Pancreatic cancer	CD133^+,^ CD44^+^, CD24^+^, ABCG2, ALDH, EpCAM^+^, ESA	[195, 215, 218]
Melanoma	ABCB5^+^, CD20^+^	[51, 217]
Head and neck cancer	SSEA-1^+^, CD44^+^, CD133^+^	[224–226]
